# Fanconi–Bickel Syndrome: A Review of the Mechanisms That Lead to Dysglycaemia

**DOI:** 10.3390/ijms21176286

**Published:** 2020-08-31

**Authors:** Sanaa Sharari, Mohamad Abou-Alloul, Khalid Hussain, Faiyaz Ahmad Khan

**Affiliations:** 1 Division of Biological and Biomedical Sciences, College of Health & Life Sciences, Hamad Bin Khalifa University, Qatar Foundation, Education City, Doha, Qatar; sansharari@hbku.edu.qa; 2 Department of Pediatric Medicine, Division of Endocrinology, Sidra Medicine, Doha, Qatar; khussain@sidra.org; 3 Department of Pediatric Medicine, Saida Governmental University Hospital, Beirut Arab University, Beirut 115020, Lebanon; m.aboaloul@hotmail.com

**Keywords:** Fanconi–Bickel Syndrome (FBS), *SLC2A2* mutation, GLUT2 dysfunction, dysglycaemia, liver, pancreatic β cell, cAMP, insulin secretion, birth weight, hepatomegaly

## Abstract

Accumulation of glycogen in the kidney and liver is the main feature of Fanconi–Bickel Syndrome (FBS), a rare disorder of carbohydrate metabolism inherited in an autosomal recessive manner due to *SLC2A2* gene mutations. Missense, nonsense, frame-shift (fs), in-frame indels, splice site, and compound heterozygous variants have all been identified in *SLC2A2* gene of FBS cases. Approximately 144 FBS cases with 70 different *SLC2A2* gene variants have been reported so far. *SLC2A2* encodes for glucose transporter 2 (GLUT2) a low affinity facilitative transporter of glucose mainly expressed in tissues playing important roles in glucose homeostasis, such as renal tubular cells, enterocytes, pancreatic β-cells, hepatocytes and discrete regions of the brain. Dysfunctional mutations and decreased GLUT2 expression leads to dysglycaemia (fasting hypoglycemia, postprandial hyperglycemia, glucose intolerance, and rarely diabetes mellitus), hepatomegaly, galactose intolerance, rickets, and poor growth. The molecular mechanisms of dysglycaemia in FBS are still not clearly understood. In this review, we discuss the physiological roles of GLUT2 and the pathophysiology of mutants, highlight all of the previously reported *SLC2A2* mutations associated with dysglycaemia, and review the potential molecular mechanisms leading to dysglycaemia and diabetes mellitus in FBS patients.

## 1. Introduction

Fanconi–Bickel syndrome (OMIM# 227810), a carbohydrate metabolism disorder due to glucose transporter 2 (GLUT2) transporter defect was first described by Fanconi and Bickel in 1949 [1]. In 1987, Fanconi–Bickel Syndrome (FBS) was identified as a defect in a sodium glucose secondary active transporter responsible for galactose and glucose transport in many tissues, including kidney and liver [2]. Studies in 1989, using Xenopus oocytes injected with human liver type glucose transporter synthetic mRNA construct, identified the role of the plasma membrane glucose transporter in sensing glucose, uptake of glucose, and its possible role in non-insulin-dependent diabetes mellitus [3]. In 1994, a study in Xenopus oocytes proved that a highly conserved GLUT2 missense mutation in one allele of the gene (substituted Val197 to Ile197) leads to GLUT2 dysfunction, and might be expected to play an important role in pathogenicity of non-insulin dependent diabetes mellitus [4,5]. Then in 1997, Santer et. al. (1997) for first time described the role of GLUT2 (*SLC2A2*) gene mutations in three FBS affected families, that includes the original patient reported by Fanconi and Bickel in 1949 [6].

FBS is a rare disorder of carbohydrate metabolism with phenotypic variability in patients and is inherited in an autosomal recessive manner. FBS is due to abnormal accumulation of glycogen in liver and kidneys leading to impairment of glucose and galactose utilization, and proximal renal tubular dysfunction [2]. The clinical features in FBS patients include short stature, rickets, failure to thrive, hepatomegaly, polyuria, proximal renal tubular dysfunction, and osteoporosis [2,7,8,9,10]. Biochemically, in glucose physiology, FBS patients have dysglycaemia characterized by fasting hypoglycemia, post-prandial hyperglycemia, glucose intolerance, and rarely diabetes mellitus. The pathology of FBS is due to compound heterozygous mutation (each allele with different mutation) or homozygous mutations in the GLUT2 (*SLC2A2)* gene [4,6,11,12]. This coding gene contains 11 exons and 10 introns, located on chromosome 3q26.1–q26.3. Human GLUT2 consists of 524 amino acids (aa) and the transmembrane segment 9 to 12 play a vital role in the characteristic affinity for glucose [13,14,15]. As discussed above, GLUT2 is mainly expressed in hepatocytes, enterocytes [16], proximal tubules in the kidney [17], pancreatic β-cells [15,18,19], discrete neuronal cells [20], and in astrocytes [21].

Dysglycaemia (fasting hypoglycemia, postprandial hyperglycemia, glucose intolerance, and diabetes mellitus) is commonly observed in many patients with FBS, however the mechanisms underlying dysglycaemia are not well understood. In this review, we briefly summarize the role of GLUT2 in glucose physiology, then, review all the reported mutations in *SLC2A2*, which have been reported with dysglycaemia and we finally discuss the potential molecular mechanisms of dysglycaemia associated with FBS.

## 2. Physiological Roles of GLUT2

### 2.1. Overview

GLUT2 has a low affinity (high *K*_M_) for glucose (15–20 mM) and plays a role in organs that have an important function in controlling glucose homeostasis such as the pancreatic β-cells, kidney, intestine, and liver. GLUT2 maintains glucose homeostasis by regulating the transepithelial uptake of glucose in the epithelial cells of basolateral membrane, glucose reabsorption in the kidney proximal tubule, glucose uptake and release from the sinusoidal membrane of the liver cells, and glucose regulated insulin secretion from pancreatic β-cells [22]. For more comprehensive reviews on the physiological roles of GLUT2, please see references [23,24,25].

### 2.2. Role of GLUT2 in β-Cells 

GLUT2 function in human pancreatic β-cells is not clearly understood and is still controversial. However, the role of GLUT2 in rodent pancreatic β-cells is well established and plays a major role in glucose transport [26]. Expression of glucose transporters in rodent β-cells is different from human β-cells, with human β-cells showing predominantly GLUT1 and GLUT3 instead of GLUT2 expression [27]. Rat and human pancreatic β-cells have similar gene expression of glucokinase, but different expression of glucose transporters [27]. There is an abundance of GLUT1 transcript and protein expression in human β-cells and the glucose transporter kinetic characteristics are similar to GLUT1 [15]. During human pancreatic development, both GLUT1 and GLUT2 are expressed in the fetal islets [28], with GLUT1 reaching adult transcriptional levels by 18 weeks of gestational age and GLUT2 mRNA is detectable as early as 13 weeks of gestational age. However, both GLUT1 and GLUT2 expression remains low throughout human pancreatic development in the β-cells [29].

In rodents, GLUT2 might also have a role in pancreatic β-cell glucose sensing independent from its role as a glucose transporter. One study, using liver cells which were transfected with GLUT2-intracellular loop amino acids (aa) 237–301 fused with GFP (green fluorescent protein) cDNA shows translocation of the GLUT2-GFP fused protein to the nucleus from the cytoplasm at high concentration of glucose. This suggests that, via protein interactions with its large loop, GLUT2 may transduce a glucose signal from the plasma membrane to the nucleus [30]. In another study, transgenic mice expressing the same fusion protein had multiple defects of glucose metabolism [31]. These mice also had greater amounts of urine glucose, which might play an important role in protecting the mice from developing hyperglycemia despite the reduced production of insulin from the β-cells.

**Figure 1:**
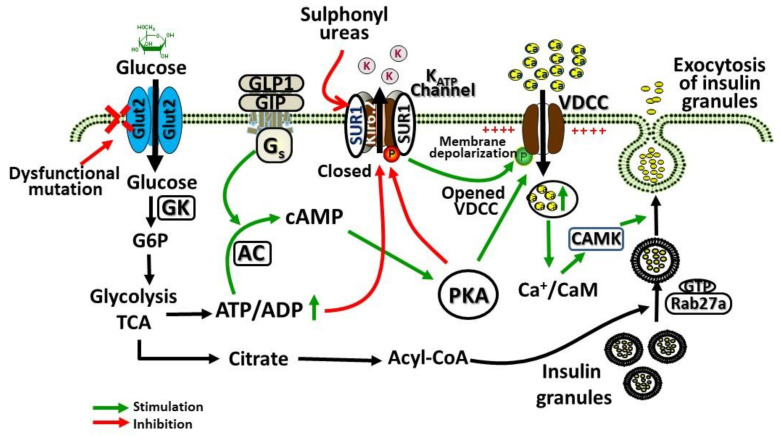
Role of glucose transporter 2 (GLUT2) in pancreatic β cell glucose-stimulated insulin secretion. Glucose enters the β-cells facilitated by GLUT2, which is then phosphorylated by glucokinase (GK) and metabolized by glycolysis and tricarboxylic acid cycle (TCA), leading to increased generation of ATP. Glucagon like peptide-1 (GLP1), glucagon, and GIP (gastric inhibitory polypeptide) activation of G protein-coupled receptors (GPCRs) responsive adenylyl cyclases (AC), leading to increased formation of cyclic adenosine monophosphate (cAMP). cAMP activates protein kinase A (PKA) and epac2 (cAMP-regulated guanine nucleotide exchange factor) to potentiate glucose-stimulated secretion of insulin. Increased ratio of ATP/ADP due to increased glucose metabolism closes the ATP-gated K+ channel (K_ATP_), causing membrane depolarization, and voltage-dependent calcium channels (VDCC) opening, leading to Ca^2+^ influx via VDCC, which activates insulin secretion. This action of Ca^2+^ is potentiated by PKA. For exocytosis of insulin from β-cells, Rab3a and Rab27a GTPases are important signaling mediators that become activated by this mechanism and contribute to the exocytosis of insulin from glucose-stimulated cells.

GLUT2 is a high *K*_M_ (15–20 mM) and low affinity facilitative transporter of glucose allowing the glucose transport in proportion to the circulating blood glucose levels. This ensures the sensing and appropriate responses by the liver and pancreas for controlling the normal blood glucose levels [32]. GLUT2 is presumed to play a key role in regulating the molecular mechanisms of glucose-stimulated secretion of insulin. This molecular mechanism involves the transport of glucose through GLUT2 in β-cells, phosphorylation of glucose by glucokinase leading to increased glycolysis and production of ATP. An increased ratio of ATP/ADP leads to the potassium channel (K_ATP_) closure and inhibition of potassium efflux, leading to β-cell membrane depolarization, opening of the calcium channel, facilitating calcium entry inside the cell, and causing insulin secretion. Figure 1 shows the regulation of pancreatic β cell glucose-stimulated insulin secretion by GLUT2.

### 2.3. Role of GLUT2 in Liver

**Figure 2:**
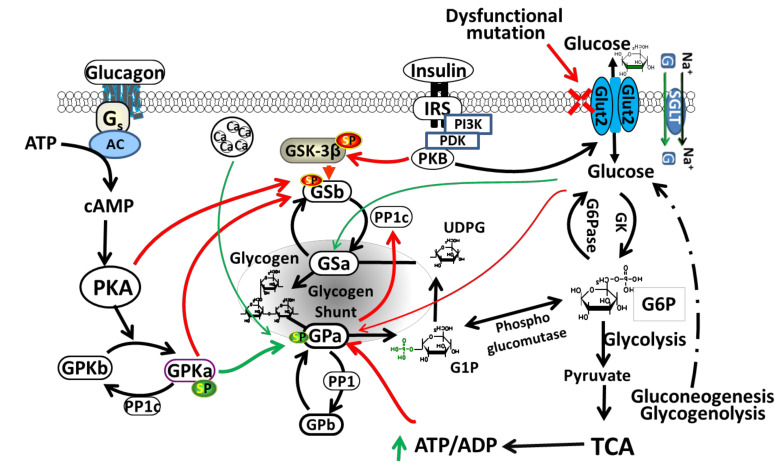
Regulation of glycogen metabolism in liver by GLUT2. The insulin-independent GLUT2 facilitates glucose transport across the cell membranes. Sodium-glucose cotransporter (SGLT) transport activity can be regulated by protein kinases including PKA (protein kinase A) and PKC (protein kinase C). PKA activation positively regulates SGLT1 expression and activity. Increased glucose concentration causes conformational change and activation of glucokinase (GK), which phosphorylates glucose to glucose-6-phosphate (G6P) and serves as a substrate for glycogen synthesis or glycolysis. In hepatic glycogen metabolism, G6P serves as the central intermediate. During glycolysis, utilization of G6P provides energy in the form of pyruvate, NADH and ATP. Acetyl-CoA produced from pyruvate, enters the mitochondrial tricarboxylic acid cycle (TCA). During fasting, G6P serves as a substrate for synthesis of glucose during gluconeogenesis or glycogenolysis. Glycogen phosphorylase (GP) catalyze glycogenolysis, activated by AMP or PKA, and inhibited by insulin. GSK-3β (a serine/threonine kinase) is a downstream element of PI3K/AKT pathway, whose activity can be inhibited by protein kinase B (PKB)-mediated phosphorylation at Ser9 of GSK-3β. Glycogen synthase (GS) is phosphorylated and inactivated by GSK3. SGLT: sodium-glucose cotransporter; G: glucose. Green arrows (stimulation); Red arrows (inhibition); Green Sp (phosphorylated serine): activating phosphorylation; Red Sp: inhibiting phosphorylation. Dashed lines indicate many steps not listed in the gluconeogenesis and glycogenolysis pathways to glucose.

Liver GLUT2 plays an important role in homeostasis of glucose by regulating glucose uptake and release, depending on a fasting or feeding state [24,33]. In rodents and humans, GLUT2 is considered the major transporter of glucose in liver [11,32,33]. It is located in the basolateral (sinusoidal) plasma membrane domain of hepatocytes [18]. GLUT2 in the liver regulates glucose release (produced either from gluconeogenesis or glycogenolysis) during the fasting state and glucose uptake during the feeding state leading to glycogen synthesis in the liver [24,34]. Glycogen synthesis occurs through glycogen synthase (GS) (glycogen synthesis key enzyme) activation by allosteric stimulator phosphorylated glucose (glucose-6-phosphate) and through inactivation of glycogen synthase kinase-3 (GSK-3) with insulin, however glycogenolysis is inhibited by activation of glycogen phosphorylase kinase [35]. Figure 2 shows the regulation of glycogen metabolism in liver by GLUT2. During hyperglycemia, GLUT2 facilitates glucose transport across the cell membranes leading to activation of glycogen synthesis pathway. However, in hypoglycemia, glycogen phosphorylase (GP) catalyzes glycogenolysis, activated by AMP or protein kinase A (PKA).

The inactivation of GLUT2 in adult mice hepatocytes provides some interesting information about the role of GLUT2 in the physiology of glucose metabolism [36]. In these mice, the liver size is 40% larger due to increased glycogen storage, and fasting did not reverse the phenotype [37]. The loss of GLUT2 leads to reduced hepatic glucose uptake without any change in glucose output in these mice with normal hepatic glucose production suggesting glucose being produced in hepatocytes may use an alternative pathway to be released independent of GLUT2 or any facilitative diffusion at the plasma membrane [38,39]. When these mice were fed, there was an elevated expression of lipogenic and glycolytic target genes and ChREBP (Carbohydrate-Response Element-Binding Protein), which regulates transcription of genes related to lipid and glucose metabolism. More interestingly, both GLUT2 hepatocyte knockout mice and control mice had normal expenditure of energy, feeding behavior, and sensitivity of insulin. There was an increased decline in glucose-stimulated insulin secretion even without any changes in the contents of insulin and β-cell mass in the GLUT2 knockout mice hepatocyte.

GLUT2 mRNA and protein expression in hepatocytes is known to be modulated by thyroid hormones. In crude liver membranes, GLUT2 protein expression was found to be significantly increased in chronically hyperthyroid compared to hypothyroid animals [40]. However, total GLUT2 protein was similarly expressed when assayed in detergent extracts of liver. These observations suggest that glycolysis and gluconeogenesis enzymes together with the GLUT2 glucose transporter expression and functions are target for hormonal regulation that control hepatic glucose metabolism. GLUT2 also forms a complex with the insulin receptor forming a receptor-transporter complex providing a direct pathway between insulin binding and glucose transport regulation [41]. Protein Tyrosine Phosphatase-1B (PTP-1B) deficient mice studies suggested that GLUT2 and insulin receptor complex plays a vital role and modulate glucose uptake in neonatal mice hepatocytes [42]. Given the key role of GLUT2 in the liver, it explains some of the abnormalities observed in glucose metabolism in FBS patients (fasting hypoglycemia, postprandial hyperglycemia, and glycogen storage).

### 2.4. Role of GLUT2 in Kidney 

Three glucose transporters (sodium-glucose cotransporter 1 (SGLT1), SGLT2, and GLUT2) play important roles for reabsorption of glucose in the proximal tubule of the kidney from the glomerular filtrate. In the kidney, glucose transporters GLUT2 is localized in the basolateral membrane; SGLT1 (sodium−glucose cotransporter-1) and SGLT2 are localized in the apical membrane [43]. Bulk of the filtered glucose is reabsorbed by SGLT2, and SGLT1 plays a role as reserve capacity for the filtration of glucose. In the basolateral membrane, GLUT2 plays an essential role for completing the glucose reabsorption. In FBS patients, GLUT2 dysfunction causes glycogen accumulation in the proximal tubules of the kidney [44]. GLUT2 dysfunction in proximal renal tubules leads to glycosuria, metabolic acidosis, phosphaturia, hypercalciuria, generalized aminoaciduria, bicarbonaturia, hypophosphatemia, and nephropathy [8,45,46,47]. Recent reports suggest that the expression of GLUT2 is upregulated in the renal proximal tubules during diabetes [48].

### 2.5. Role of GLUT2 in Intestine

The rate of movement of the contents of stomach (gastric emptying) and the absorption of glucose in the intestine regulate the glucose appearance rate in the circulation. Enterocytes in the small intestine are polarized epithelial cells and play important roles in the absorption of nutrients and uptake of glucose using various transporters to the blood vessels. This is the primary mechanism for the transport of glucose and other nutrients from intestine enter into the blood circulation. Two glucose transporters are expressed on the enterocytes, namely SGLT1 (sodium glucose co-transporter 1) and GLUT [49]. The GLUT2 transporters are situated in the enterocyte basolateral membrane, while SGLT1 is located in the intestinal membrane brush border. SGLT1 transports luminal glucose across the basolateral membrane and into the enterocyte by an active process. Once inside the enterocytes, glucose is phosphorylated to glucose-6-phosphate (G6P) by hexokinase and accumulates, G6P is dephosphorylated by glucose-6-phospatase to glucose for transport to portal vein. Dephosphorylated glucose is then passively transported out of the enterocytes by GLUT2 in the basolateral membrane. In conditions of high glucose load in the intestine endosomal GLUT2 can be rapidly translocated to the apical membrane to facilitate increased glucose absorption [34]. Interestingly, glucose absorption from the rat intestine can be enhanced by artificial sweeteners due to increased expression of apical GLUT2 [50]. Intestinal GLUT2 is a facilitative glucose transporter and also has the ability to transport galactose, mannose, glucosamine, and fructose [51].

### 2.6. Role of GLUT2 in Brain

Glucose is transported across the blood brain barrier facilitated by glucose transporters (GLUTs), since neurons in the brain are not able to store or synthesize glucose. GLUT1 and GLUT3 are the main glucose transporters in the brain. GLUT1 is highly expressed in the blood brain barrier endothelial cells and transports glucose to the brain extracellular space from the blood. GLUT3 is the main glucose transporter into the neuronal cells from the extracellular space, and in astrocytes GLUT2 is main glucose transporter. Apart from transporting glucose into the astrocytes, GLUT2 plays a role as a glucose sensor in the brain. In GLUT2 knockout mice, studies have shown that GLUT2 elimination leads to suppression of glucose sensing in the brain and this impacts feeding behavior probably by regulating the hypothalamic melanocortin pathways [52]. GLUT2, via the autonomic nervous system also links brain glucose sensing with pancreatic β-cell mass and function. Nervous system GLUT2 inactivation in mice leads to the late onset glucose intolerance development with reduced secretion of insulin due to reduced β-cell mass and proliferation [53]. Both parasympathetic and sympathetic activity were reduced and not affected by glucose. Thus GLUT2 plays an important role in linking the endocrine pancreas with the central nervous system.

## 3. *SLC2A2* (GLUT2) Mutations and Patterns of Dysglycaemia

**Figure 3:**
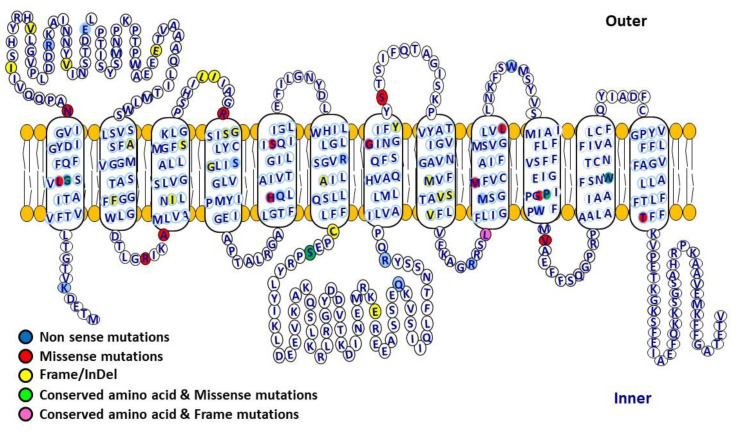
Human glucose transporter 2 (GLUT2) structural topology including location of variants and conserved amino acids. GLUT2 is a glucose transporter containing 12 transmembrane domains (524 amino acids) connected with extracellular and intracellular loops, facilitating the movement of glucose across the cell membranes. It has high capacity but low affinity (high km) for glucose, and functions as a glucose sensor. GLUT2 mediates bidirectional glucose transport, and is mostly expressed in pancreatic β-cells, liver, small intestine, brain and renal tubular cells.

From 1987 to the present, 144 clinical reports of FBS have been published. All the FBS published cases have been summarized in Table 1, including those with and without mutations in the *SLC2A2* gene. Table 2 and Figure 3 list all of the different types of mutations that are reported in the *SLC2A2* gene. These mutations included missense, nonsense, fs/indel, intronic and compound heterozygous mutations. We analyzed the pattern and severity of dysglycaemia associated with different types of *SLC2A2* gene mutations. The patterns of dysglycaemia and the mutations are shown in Table 3. In this table, we only included those cases where there was a clear clinical description of the patient having blood glucose measurements to document either fasting hypoglycemia, post-prandial hyperglycemia, glucose intolerance or frank diabetes. The pattern of dysglycaemia ranged from fasting hypoglycemia, post-prandial hyperglycemia, glucose intolerance, transient neonatal diabetes to gestational diabetes and frank diabetes mellitus. There was no correlation between the mutation type and the pattern of dysglycaemia. We also analyzed the birth weight of the FBS patients with GLUT2 mutations and dysglycaemia, and these are shown in Table 4. Virtually all patients with GLUT2 mutations had low birth weights.

### 3.1. Potential Biochemical Mechanisms Leading to Dysglycaemia in Patients with FBS 

Dysglycaemia is defined as any abnormality in the level of blood glucose including hypoglycemia or hyperglycemia. Patients with FBS show several different patterns in the blood glucose levels, including fasting hypoglycemia, post-prandial hyperglycemia, impaired glucose tolerance, transient neonatal diabetes, and very rarely late onset diabetes mellitus. The underlying biochemical and molecular mechanisms that lead to these blood glucose profiles are not well understood. Homozygous GLUT2 knockout mice demonstrate hyperglycemia with relative hypoinsulinism, develop frank diabetes mellitus and die two to three weeks after birth [105]. These mice lack the first-phase secretion of insulin which is pancreatic K_ATP_ channels dependent, but the second-phase secretion of insulin is preserved which is dependent on amplifying pathways [105]. During hyperglycemia, pancreatic β-cells are exposed to higher glucose concentrations which amplifies the glucose enhanced signal of insulin secretion through exocytosis of insulin granules in a time-dependent manner. This observation indicated that GLUT2 is required for the first phase of insulin secretion. In addition, the mice have elevated serum glucagon levels and ketone bodies (β-hydroxybutyrate). At a histological level, GLUT2 deletion in these mice leads to the postnatal pancreatic islets developmental changes as shown by a change in pancreatic alpha- to β-cell ratio. 

### 3.2. Birth Weight in FBS

In contrast to homozygous GLUT2 knockout mice, frank diabetes mellitus is rare in FBS patients. Sansbury et al. [74,106] first reported transient neonatal diabetes mellitus (TNDM) in four FBS patients due to homozygous *SLC2A2* mutation with loss of function which resolved at a median 18 months of age, and fifth patient was still on the treatment of insulin at the age of 28 months. All five of these patients had low birth weight which suggests a defect in β-cell insulin secretion in the last trimester of pregnancy, as insulin is a growth factor. The low birth weight is an interesting observation as we found that virtually all FBS patients are born with low birth weights (Table 4). Two of the reported patients by Sansbury et al. had a relatively low C-peptide level at the time of diagnosis, again suggesting defects in β-cell function. All patients needed treatment with subcutaneous insulin to control their diabetes mellitus. 

### 3.3. Neonatal Diabetes in FBS

Since the initial report of transient neonatal diabetes by Sansbury et al. [74,106], two more cases have been described with homozygous *SLC2A2* gene mutations. Yoo et al. (2002) reported a newborn with transient neonatal diabetes and galactosemia with a novel *SLC2A2* homozygous gene mutation [83], and Khandelwal et al. (2018) reported phenotypic heterogeneity in siblings with one of the siblings having transient neonatal diabetes [55]. The findings of transient neonatal diabetes in some cases of FBS suggest an important role of GLUT2 in human β-cell physiology. The loss of function mutations in *SLC2A2* presumably leads to reduced function or expression of GLUT2 in the pancreatic β-cell impairing insulin secretion, leading to hyperglycemia. After birth, there must be some compensatory mechanism(s) which allow the diabetes to remit, possibly involving GLUT1 or GLUT3. The intriguing fact is that not all cases of FBS patients develop transient neonatal diabetes mellitus and this suggests that there are other molecular mechanisms associated with the onset of the neonatal diabetes that may not be directly related to GLUT2. These studies suggest that in humans GLUT2 play important roles more likely in the neonatal and developmental period. 

To the best of our knowledge there are no cases of FBS patients reported with neonatal diabetes where pancreatic histology has been studied at post-mortem, so it is not possible to say if there are any morphological changes as observed in the GLUT2 knockout mice. All these FBS patients with transient neonatal diabetes should be followed up in the long-term to see if the diabetes relapses (as occurs with other causes of transient neonatal diabetes). There is marked interindividual heterogeneity in the expression and regulation of SGLT2 (Sodium-glucose co-transporter-2) and this could account for some of the phenotypic differences observed between individuals even in the same family [107].

### 3.4. Frank Diabetes in FBS

Frank Diabetes is very rare in older patients with FBS. Seker-Yilmaz et al. [58] studied eight patients with FBS and found that only one had significant hypoglycemia, all patients had post-prandial hyperglycemia with two patients having blood glucose levels >200 mg/dL at 120 min after the oral glucose tolerance test (which by definition is diabetes) but their hemoglobin-A1c (HBA1c) value was normal (due to the low blood glucose level). Taha et al. (2008) [101] undertook the oral glucose testing (OGTT) in ten patients with FBS to evaluate the insulin and glucose levels in response to oral glucose load. They found that most but not all patients with FBS have impaired glucose tolerance/diabetes range hyperglycemia after the OGTT while maintaining normal HbA1c. Patients with FBS were also found to be relatively hypoinsulinemic. Ganesh R. et al. [102] evaluated twelve infants with infantile onset diabetes and found that one of the patients had FBS, and Pena et al. [78] have reported an adult female at the age of 31 years with FBS who developed gestational diabetes. In this case, it is unclear if the gestational diabetes is directly linked to the GLUT2 defect or is just a co-incidence. Interestingly one particular polymorphism in the GLUT2 gene (valine 197 to isoleucine) was found in a patient with gestational diabetes which showed a loss of function mutation of GLUT2 when tested in Xenopus oocytes [4].

In some of the previous studies, no associations were found between GLUT2 gene polymorphisms and diabetes mellitus [108,109]. More recent Genome Wide Associations studies (GWAS) have found associations between GLUT2 variants and fasting hyperglycemia, glucose intolerance and type 2 diabetes mellitus [110,111], but the underlying mechanisms linking variants in GLUT2 and abnormalities in glucose physiology are not clear from these GWAS studies. Santer et al. [89] followed up the original patient with FBS who was described in 1949 after 50 years and did not report overt diabetes in that patient. This is probably the longest follow up of any FBS patient and suggests that late frank diabetes is not a clinically recognized feature of FBS. 

### 3.5. Glycogen Storage in FBS

The glycogen accumulation mechanism in FBS patients is presumably due to GLUT2 deficiency and defective glucose transport during glycogenolysis to the extracellular compartment. This causes an accumulation of intracellular glucose, leading to reduced glycogenolysis and increased glycogen storage. Impaired glucose export due to GLUT2 deficiency in hepatocytes causes fasting hypoglycemia when sources of peripheral glucose are diminished. The hypoglycemia is exacerbated by the glycosuria from the renal tubular leak. In the FBS patients due to nonfunctional GLUT2 mutation, defective uptake of monosaccharides and reduced glucose-stimulated secretion of insulin from pancreatic β-cells in the fed state causes hyperglycemia and hypergalactosemia [6]. FBS patients have relative hypoinsulinemia for the levels of blood glucose [2,101], as expected due to a defective secretion of first phase of insulin.

**Figure 4:**
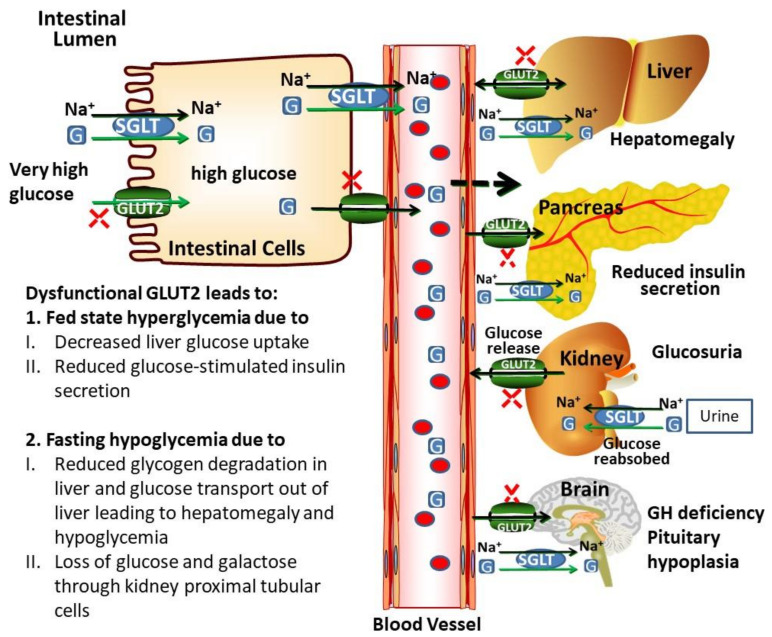
GLUT2-mediated glucose transport across cell membranes and communication to different organs. GLUT2 is a low affinity and high capacity glucose transporter and facilitates the transport of glucose in intestinal cells. SGLTs are high-affinity and low capacity transporters, capable of transporting glucose against a concentration gradient. Ghrelin increases the GLUT2 expression by modulating the GLUT2 transcription via GHS-R1 (growth hormone secretagogue receptor 1a) and PLC/PKC pathway. Ghrelin also stimulates translocation of GLUT2 to the surfaces of intestinal cell from intracellular vesicles, leading to increased glucose absorption. GLUT2 is highly expressed in the liver, pancreas, brain and kidney cells. Activation of nervous signals, induces the first phase of insulin release from pancreatic β-cells induced by increased glucose transport to pancreatic β-cells. These signals also induce other physiological processes. In the liver, GLUT2 maintains the glucose homeostasis during fasting and fed state by regulating the expression of glucose-sensitive genes. GLUT2 is more highly expressed in rodent β-cells than in human β-cells. In the brain, glucose-sensing cells expressing GLUT2, regulate glucose by parasympathetic and sympathetic systems. GLUT2-dependent glucose-sensing cells also regulate leptin sensitivity and regulate the expressions of uncoupling protein 1 (UCP1) and thermogenesis. Homozygous GLUT2 mutation leads to a dysfunctional and reduced expression of GLUT2, which causes FBS. Dysfunctional GLUT2 causes fasting hypoglycemia, postprandial hyperglycemia, glucose and galactose intolerance, hepatomegaly, glucosuria, reduced GSIS (glucose-stimulated insulin secretion), rickets, GH (growth hormone) deficiency and poor, growth. (G; glucose).

In addition, GLUT2 dysfunction in the liver can lead to hyperglycemia due to decreased response and sensitivity of hepatocytes to insulin signaling, causing a decreased inhibition of glucose production [112]. In the FBS patients, due to repeated episodes of hypoglycemia, glycated HbA1c (haemoglobin-A1c) levels are usually in the normal range. Hyperglycemia in the fed state and hypoglycemia in the fasting state appear to improve over time [101]. Glucose absorption is normal from the intestine of FBS patients suggesting that GLUT2 involvement in the facilitative glucose transport in the enterocytes must be involved at the basolateral membrane [113]. These observations are supported by studies in GLUT2 knockout mice which demonstrate the role of glucose transepithelial transport system in the mice intestine that requires phosphorylation of glucose [114]. Thus, dysglycaemia in FBS patients is not due to abnormalities in glucose absorption from the intestine. Figure 4 summarizes the expected pathophysiology of GLUT2 dysfunction based on the clinical profile of FBS: Postprandial hyperglycemia is due to an impairment of glucose transportation from the enterocytes and decreased glucose uptake by the liver due to impaired insulin secretion. Hypoglycaemia in the fasting state is due to the lack of renal glucose reabsorption from the kidney, as well as the accumulation of glucose in the liver leading to reduced glycogen breakdown.

### 3.6. Structure Function Relationship of GLUT2 in FBS

The structure-function relationships of several naturally occurring FBS *SLC2A2* mutations and several engineered mutations and their effects on kinetic parameters, protein expression, differentiation of β-cell, and secretion of insulin has been examined using Xenopus oocytes, hepatocytes, and pancreatic β cells [115]. The naturally occurring FBS *SLC2A2* mutations showed loss of transport function despite the similar to wild-type targeting of the protein on the plasma membrane and similar or reduced expression of protein. These functional changes in GLUT2 could be due to reduced capacity of glucose transport despite the proper targeting of the transport protein at the plasma membrane or due to loss of protein expression. These findings point to the vital role of GLUT2 in glucose transport. All the engineered mutants of GLUT2 are found to be localized at the plasma membrane and able to transport glucose, with modified kinetic properties and characteristics of glucose transport. In addition, they show increased β-cell differentiation and secretion of insulin. An important finding from three engineered GLUT2 transporter mutants show increased numbers of pancreatic β-cell, suggesting an important GLUT2 role in the development of pancreatic β-cells. These observations suggested a functional role for GLUT2 as an important glucose transporter and extracellular receptor, which have an effect on pancreatic β cell differentiation and secretion of insulin.

## 4. Conclusions and Future Perspective

A large number of the reported patients with FBS have dysglycaemia characterized by post-prandial hyperglycemia, fasting hypoglycemia, glucose intolerance, and rarely diabetes mellitus. The role of human GLUT2 in pancreatic β-cell physiology is still unclear, although the increasing number of cases of transient neonatal diabetes suggests that it might have a role in the newborn period. The low birth weights of virtually all the reported FBS cases suggests that GLUT2 might have a role in human fetal insulin physiology. Currently there are no drugs in clinical practice which target the underlying molecular defect in GLUT2 in patients with FBS and management of these patients is clinically challenging. Future GLUT2 functional studies in human pancreatic β-cells, liver, enterocytes, kidney, and brain cells, will be required for a better understanding of underlying molecular mechanisms of dysglycaemia in FBS that will facilitate the development of precision medicine based on the molecular mechanisms of GLUT2.

## Figures and Tables

**Table 1:** Fanconi–Bickel Syndrome (FBS) cases reported from 1987 to 2020.

Patient Number	Reference	Sex	Origin	Mutation	Amino Acid Change
1	[9]	F	Pakistani	c.497-2A > T	p.(Gly166_Ser169del)]
2	[9]	M	Pakistani	c.497-2A > T	p.(Gly166_Ser169del)]
3	[54]	F	Turkish	IVS8g.24401-24406del6	NR
4	[55]	F	India	c.952G>A	p.Gly318Arg
5	[55]	M	India	c.952G>A	p.Gly318Arg
6	[55]	F	India	c.952G>A	p.Gly318Arg
7	[56]	M	Kuwait	c.474A>C	p. Arg158Ser
8	[57]	M	Turkish	c.108+5G>A	NR
9	[58]	M	Turkish	c.1069delGinsAATAA	p. Val357AsnfsTer37
10	[58]	F	Turkish	c.482_483insC	p. Gly162ArgfsTer17
11	[58]	NR	Turkish	c.482_483insC	p. Gly162ArgfsTer17
12	[58]	M	Turkish	c.482_483insC	p. Gly162ArgfsTer17
13	[58]	NR	Turkish	c.482_483insC	p. Gly162ArgfsTer17
14	[58]	M	Turkish	c.482_483insC	p. Gly162ArgfsTer17
15	[58]	M	Turkish	c.482_483insC	p. Gly162ArgfsTer17
16	[58]	M	Turkish	c.575A>G	p. His192Arg
17	[59]	M	Iran	C.685_701del GCCATCCTTCAGTCTCT ins CAGAAAA	p.A229 QfsX19
18	[60]	M	NR	NR	E85fsX177 and G189D
19	[61]	F	Indian	c.1246G>A	p. Gly416Ser
20	[60]	M	NR	NR	NR
21	[62]	M	Kashmir	NR	NR
22	[63]	F	Indian	NR	NR
23	[64]	M	Indian?	c.16-1G>A or IVS 1-1G>A	NR
24	[65]	F	African American	c.670	Cys224Del
25	[66]	F	Palestinian	c.901C>T	p. Arg301Ter
26	[66]	M	Palestinian	c.901C>T	p. Arg301Ter
27	[67]	F	Pakistani	c.339delC	p. Phe114LeufsTer16
28	[67]	M	Pakistani	c.339delC	p. Phe114LeufsTer16
29	[68]	NR	Chinese	c.380C>A and c.970dupT	p.Ala127Asp and p.324TyrfsX392
30	[68]	NR	Chinese	c.1068+5G>C	IVS8+5G>C
31	[68]	NR	Chinese	c.1194T>A	p.Tyr398X
32	[69]	F	Iranian	c.1061_1066del6	p.V355_S356del2
33	[69]	M	Iranian	c.1061_1066del6	p.V355_S356del2
34	[70]	M	Algerian	IVS 3+2T>C/IVS 3+2T>C	NR
35	[10]	M	Indian	c.1330T>C	p. W444R
36	[71]	F	Iranian	c. 685_701del GCCATCCTTCAGTCTCTins CAGAAAA	P.A229QFsX19
37	[72]	F	Indian	c.56T>C	p. Leu19Pro
38	[73]	F	Indian	NR	NR
39	[74]	F	Chinese	c.609T>A	p.Ser203Arg
40	[74]	M	Oman	c.1127T>G	p.Met376Arg
41	[74,75]	F	Iran	c.963+1G>A	NR
42	[74]	M	Sudanese	c.157C>T	p.Arg53X
43	[74]	M	Saudi Arabia	c.339del	p.Phe114LeufsX16
44	[76]	F	Egyptian	c.1250C>T	p. P417L
45	[76]	M	Egyptian	c.253_254del GA	p. Glu85fs
46	[77]	M	Egyptian	c.776-1G>C	NR
47	[78]	F	Caucasian	c.1439C>G and c.1469delA	T480R and L490SfsX24
48	[79]	M	Turkish	c.835_836delGA	p.E279KfsX6
49	[80]	F	NR	c.1213C>T	NR
50	[81]	M	Turkish	783del17	NR
51	[81]	F	Turkish	c.818C>G	NR
52	[81]	F	Turkish	IVS5+1 G>T	NR
53	[82]	M	Italian	425_7/delTAA	NR
54	[83]	F	Korean	c.322A>T	K5X
55	[84]	F	Japanese	c.96T>G	N32K
56	[85]	M	Japanese	c.1093 C>T and c.1642 T>C	p. Arg365Ter and p. Trp444Arg
57	[86]	F	Japanese	nt 1580T>A	V423E
58	[86]	M	Japanese	IVS2–2A>G	NR
59	[86]	M	Japanese	c.1171C>T and c.1478T>C	Q287X and L389P
60	[87]	M	Japanese	c.1159G>A	W420X
61	[87]	F	Japanese	NR	NR
62	[88]	M	Saudi-Arabian	c.1250 C>T	Pro417Leu
63		NR	Arabian	c.1250C>T	NR
64		NR	Arabian	c.1250 C>T	NR
65		NR	Arabian	c.1250 C>T	NR
66		NR	Arabian	c.1250 C>T	NR
67		NR	Arabian	c.1250 C>T	NR
68	[6,89]	M	Swiss	c.1251C>T or 1213 C>T	R301X
69	[6]	F	NR	ΔT446-449	
70	[6]	M	NR	ΔT446-449	
71	[6]	M	NR	c.1405C>T	R365X
72	[90]	M	Japanese	c.1171C>T and c.1478 T>C	NR
73	[91]	M	Caucasian	NR	NR
74	[91]	M	Caucasian	NR	NR
75	[4]	NR	Japanese	c.1571G>A	NR
76	[2]	F	Arabian	c.1562C>T	NR
77		M	Arabian	c.1562C>T	NR
78		M	Arabian	c.1562C>T	NR
79		NR	Arabian	c.1562C>T	NR
80		NR	Arabian	c.1562C>T	NR
81		NR	Arabian	c.1562C>T	NR
82		NR	Arabian	c.1562C>T	NR
83		NR	Arabian	c.1562C>T	NR
84		NR	Arabian	c.1562C>T	NR
85	[92]	M	Iran	IVS8+1 G>T	NR
86	[93]	M	Caucasian	c.457_462delCTTATA and c.1250C>G	p.153_4delLI and p.P417R
87	[93]	F	Caucasian	c.457_462delCTTATA and c.1250C>G	p.153_4delLI and p.P417R
88	[45]	M	Dominican Republic	IVS 4-2A>G	p. Gln166AspfsTer4
89	[45]	M	Dominican Republic	IVS 4-2A>G	p. Gln166AspfsTer4
90	[45]	F	Israeli	c.372A>C	p. Arg124Ser
91	[45]	M	Israeli	c.372A>C	p. Arg124Ser
92	[45]	F	Israeli	c.372A>C	p. Arg124Ser
93	[94]	F	Chinese Han	c.682C>T and c.1185 G>A	p. Arg228X and p. Trp395X
94	[94]	M	Chinese Han/Yao	c.196G>T and c.1117delA	p. Glu66X and p. Met373X
95	[95]	M	NR	NR	NR
96	[80]	F	NR	c.1213C>T	NR
97	[96]	F	Indian	NR	NR
98	[97]	M	Arab	c.1213C>T	p.Phe405>Leu
99	[98]	F	Turkish	NR	NR
100	[99]	F	German	NR	NR
101		M	German	627 delAGTTGGTGins GT	NR
102		M	Turkish-Assyrian	793–4 ins C	NR
103		F	Turkish-Assyrian	793–4 ins C	NR
104		M	German	NR	NR
105		M	German	NR	NR
106		M	Italian (?)	c.1213C>T	NR
107		F	Italian (?)	c.889C>T	NR
108		M	English	1363 del G and 1405C>T	NR
109		M	English	1364 del G and 1405C>T	NR
110		M	Caucasian	1264 G>A and 469C>T	NR
111		M	Turkish	449 del T	NR
112		F	Turkish	450 del T	NR
113		M	Turkish	c.1405C>T	NR
114		M	Caucasian	c.1405C>T and 1008 ins A	NR
115		M	Arabian	c.1213C>T	NR
116		M	Polish (?)	NR	NR
117		F	Polish (?)	c.469C>T	NR
118		F	Polish (?)	NR	NR
119		M	Algerian	IVS 6 +1 G>C	NR
120		M	Moroccan	1288–9TC>AA	NR
121		F	NR	c.1562C>T and IVS 8 +	NR
122				1 G>A	NR
123		M	Algerian	IVS 5 +5 G>C	NR
124		M	Algerian	IVS 5 +5 G>C	NR
125		F	French (?)	1573 ins GT and 1751C>G	NR
126		M	French (?)	1574 ins GT and 1751C>G	NR
127		F	Italian	1264G>A	NR
128		F	Italian	371G>A and 1751C>G	NR
129		M	Turkish	1562C>T	NR
130		F	NR	IVS 6 +1 g>a	NR
131		M	NR	NR	NR
132		M	NR	NR	NR
133		F	French-Canadian	494 ins 26 and 1751C>G	NR
134		F	Eskimo	1415–6 del TC	NR
135		M	Arabian	1213C>T	NR
136		F	NR	IVS 8 +1 G>A	NR
137		F	NR	NR	NR
138		F	Turkish-Assyrian	793–4 ins C	NR
139		F	Greek	712–3 del CT	NR
140		M	Algerian	IVS 3 +2 T>C	NR
141		M	NR	1092C>A	NR
142		M	Turkish	738 del 17	NR
143		F	Turkish	IVS 5 +1 G>T	NR
144	[100]	M	Japanese	c.1405C>T and c.1642T>C	NR

NR: not reported.

**Table 2:** Summary of reported seventy different *SLC2A2* mutations (Missense, Nonsense, fs/indel, Intronic, and Compound Heterozygous Mutations).

Missense Mutations	Nonsense Mutations	fs/indel Mutations	Intronic Mutations	Compound Heterozygous Mutations
G20E	K5X	I39	c.497-2A>T	p.Ala127Asp and p.324TyrfsX392
N32K	R53X	L153_I154	c.108+5G>A	p. Arg365Ter and p. Trp444Arg
R158S	E66X	C239	IVS 3+2T>C	Q287X and L389P
S203R	S169X	V355_S356	c.963+1G>A	Gly20Glu and T480R
S242R	Q193X	V45	c.776-1G>C	T480R and L490SfsX24
G318R	R228X	V60	IVS5+1 G>T	p.153_4delLI and p.P417R
S326K	Q287X	A105	IVS 2 - 2 A>G	E85fsX177 and G189D
M376R	R301X	I133	IVS4+1G>A	p. Arg228X and p. Trp395X
L389P	R365X	S145	IVS 8+1 G>T	p. Glu66X and p. Met373X
G416S	W420X	S161	IVS 5-1 G>A	
P417L		M350	c.16-1G>A or IVS 1-1G>A	
P417R		L368	IVS8g.24401-24406del6	
V423E		W420	c.1068+5 G>C	
W444R		Glu85fs		
T480R		E279KfsX6		
His192Arg		Val357AsnfsTer37		
Arg124Ser		Gly162ArgfsTer17		
Leu19Pro **		A229QFsX19		
p.Phe405>Leu		Phe114LeufsX16		
		Gln166AspfsTer4		
		Cys224Del		

**Table 3:** All of the *SLC2A2* gene mutations where dysglycaemia associated with FBS has been reported.

Type of Dysglycaemia	Mutation	Amino Acid Change	Reference
**Transient neonatal diabetes**	c.952G>A	p.Gly318Arg	[55]
	c.609 T>A	p.Ser203Arg	[74]
	c.1127 T>G	p.Met376Arg	[74]
	c.963+1G>A	NR	[74]
	c.157C>T	p.Arg53X	[74]
	c.339del	p.Phe114LeufsX16	[74]
	322 A>T	K5X	[83]
**Glucose intolerance/diabetes mellitus**	c.482_483insC	p. Gly162ArgfsTer17	[58]
	c.575A>G	p. His192Arg	[58]
	c.56 T>C	p. Leu19Pro **	[72]
**Gestational diabetes**	c.1439C>G and c.1469delA	T480R and L490SfsX24	[78]
	NR	valine 197 to isoleucine	[4]
	Other: 2 patients [101], and 1 patient [102]		
**Fasting hypoglycemia**	c.108+5G>A	NR	[57]
	783del17	NR	[81]
	818C>G	NR	[81]
	IVS5+1 G>T	NR	[81]
	c.1580T>A	V423E	[86]
	IVS 2 - 2 A>G	NR	[86]
	c.952G>A	p.Gly318Arg	[55]
	NR	E85fsX177 and G189D	[60]
	NR	NR	[62]
	NR	NR	[91]
	NR	NR	[103]
	c.1246 G>A	p. Gly416Ser	[61]
	c.339delC	p. Phe114LeufsTer16	[67]
	c.339delC	p. Phe114LeufsTer16	[67]
**Post-prandial hyperglycemia**	c.1061_1066del6	p.V355_S356del2	[69]
	IVS8g.24401-24406del6	NR	[54]
	NR	NR	[73]
	NR	NR	[96]
**Fasting hypoglycemia and postprandial hyperglycemia**	c.1069delGinsAATAA	p. Val357AsnfsTer37	[58]
	c.482_483insC	p. Gly162ArgfsTer17	[58]
	c.482_483insC	p. Gly162ArgfsTer17	[58]
	c.482_483insC	p. Gly162ArgfsTer17	[58]
	c.482_483insC	p. Gly162ArgfsTer17	[58]
	c.482_483insC	p. Gly162ArgfsTer17	[58]
	c.901C > T	p. Arg301Ter	[66]
	c.16-1G>A or IVS 1-1G>A		[64]
	c.380C>A and c.970dupT	p.Ala127Asp and p.324TyrfsX392	[68]
	c.1068+5 G>C	IVS8+5G>C	[68]
	c.1194T>A	p.Tyr398X	[68]
	c.1250C>T	p. P417L	[76]
	IVS 3+2t>c/IVS 3+2t>c)	NR	[70]
	c. 685_70l del GCCATCCTTCAGTCTCTins CAGAAAA	P.A229QFsX19	[71]
	c.253_254delGA	p. Glu85fs	[77]
	c.776-1G>C	NR	[77]
	c.835_836delGA	p.E279KfsX6	[79]
	C1213T	NR	[80]
	96T>G	N32K	[84]
	c.1171C>T and c.1478T>C	Q287X and L389P	[86]
	c.1213C>T	R301X	[89]
	ΔT446-449		[6]
	ΔT446-449		[6]
	C1405T	R365X	[6]
	c.1213 C>T	p.Phe405>Leu	[97]
	C.685_701del GCCATCCTTCAGTCTCT ins CAGAAAA	p.A229 QfsX19	[59]
	c.670	Cys224Del	[65]
	c.682C>T and c.1185 G>A	p. Arg228X and p. Trp395X	[94]
	c.196G>T and c.1117delA	p. Glu66X and p. Met373X	[94]
	c.1330 T>C	p. W444R	[10]

NR: not reported.

**Table 4:** Summary of different types of GLUT2 mutations associated with Fanconi–Bickel Syndrome and the Patient Birth Weights.

Mutation		Birth Weight (kg)	References
DNA	Protein		
**Missense**			
c.609T>A	p.Ser203Arg	1.85	[74]
c.1127T>G	p.Met376Arg	2.5	[74]
c.952G>A	p.Gly318>Arg	2.4	[55]
c.952G>A	p.Gly318>Arg	2.8	[55]
c.952G>A	p.Gly318>Arg	2.3	[55]
c.1213C>T	p.Phe405>Leu	3.23	[97]
**Non-sense**			
c.901C>T	p.Arg301X	2.8	[66]
c.901C>T	p.Arg301X	2.2	[66]
**fs/indel**			
c.322A>T	p.Lys5>X	2	[83]
c.339delC	p.Phe114LeufsX16	2.5	[74]
c. 685_701 del GCCATCCTTCAGTCTCT ins CAGAAAA	P.A229QFsX19	2.6	[71]
c.1069delGinsAATAA	p. Val357AsnfsTer37	3	[58]
C.685_701del GCCATCCTTCAGTCTCT ins CAGAAAA	p.A229 QfsX19	2.6	[59]
c.783del17		2.6	[81]
c.670	Cys224Del	2.09	[65]
**Intronic**			
c.963+1G>A	NR	2	[74]
c.16-1G>A or IVS 1- 1G>A	NR	2.5	[64]
IVS8 g.24401-24406del6	NR	2.6	[54]
c.963+1 G>A	NR	2.05	[75]
(IVS2+5G>A[c.108+5G>A])	NR	3.25	[57]
**Compound Heterozygous**			
c.457_462delCTTATA in Exon 4 and c.1250C>G in Exon 10	(p.153_4delLI) and (p.Pro417Arg)	3.773.97	[93]
	E85fsX177 and G189D	3.0	[60]
**Undefined**			
		2.8	[62]
		2.5	[63]
		2.9	[73]
		2.1	[96]
		2.5	[95]
		2.8	[104]

## Abbreviations

GLUT2	Glucose transporter 2
cAMP	Cyclic adenosine monophosphate
FBS	Fanconi–Bickel Syndrome
HbA1c	Haemoglobin-A1c
GP	Glycogen phosphorylase
PKA	Protein kinase A
G6P	Glucose-6-phosphate
GK	Glucokinase
G	Glucose
NR	Not Reported
aa	amino acid
